# Hypersensitivity of Primordial Germ Cells to Compromised Replication-Associated DNA Repair Involves ATM-p53-p21 Signaling

**DOI:** 10.1371/journal.pgen.1004471

**Published:** 2014-07-10

**Authors:** Yunhai Luo, Suzanne A. Hartford, Ruizhu Zeng, Teresa L. Southard, Naoko Shima, John C. Schimenti

**Affiliations:** Department of Biomedical Sciences, Cornell University, Ithaca, New York, United States of America; University of Washington School of Medicine, United States of America

## Abstract

Genome maintenance in germ cells is critical for fertility and the stable propagation of species. While mechanisms of meiotic DNA repair and chromosome behavior are well-characterized, the same is not true for primordial germ cells (PGCs), which arise and propagate during very early stages of mammalian development. Fanconi anemia (FA), a genomic instability syndrome that includes hypogonadism and testicular failure phenotypes, is caused by mutations in genes encoding a complex of proteins involved in repair of DNA lesions associated with DNA replication. The signaling mechanisms underlying hypogonadism and testicular failure in FA patients or mouse models are unknown. We conducted genetic studies to show that hypogonadism of *Fancm* mutant mice is a result of reduced proliferation, but not apoptosis, of PGCs, resulting in reduced germ cells in neonates of both sexes. Progressive loss of germ cells in adult males also occurs, overlaid with an elevated level of meiotic DNA damage. Genetic studies indicated that ATM-p53-p21 signaling is partially responsible for the germ cell deficiency.

## Author Summary

The precursors to sperm and eggs begin are a group of <100 cells in the embryo, called primordial germ cells (PGCs). They migrate in the primitive embryo to the location of the future gonads, then undergo a rapid proliferation over the next few days to a population of many thousands. Because these cells contain the precious genetic information for our offspring, and the DNA replication associated with rapid PGC proliferation is subject to spontaneous errors, mechanisms exist to avoid propagation of mutations. A manifestation of this is the high sensitivity of PGCs to genetic perturbations affecting DNA repair. We studied mice defective for a gene called Fanconi anemia M (*Fancm*) that is important for repair of DNA damage that occurs during replication. Although it is expressed in all tissues, only the PGCs are affected in mutants, and are reduced in number. We find that PGCs lacking *Fancm* respond by slowing cell division, and identified the genetic pathway responsible for this protective response.

## Introduction

Fanconi anemia (FA) is a genomic instability (GIN) syndrome characterized by developmental abnormalities affecting the renal, gastrointestinal and reproductive systems, the skeleton, skin pigmentation, and heart. It also causes progressive bone marrow failure and increased incidence of cancer [1], [2]. It can be caused by germline mutations in any of at least 17 genes (*FANCA, FANCB, FANCC, FANCD1(BRCA2), FANCD2, FANCE, FANCF, FANCG, FANCI, FANCJ, FANCL, FANCM, FANCN (PALB2), FANCO(RAD51C), FANCP(SLX4), FANCQ(ERCC1 or 4)*) [3], [4]). The products of these genes coordinately function in the repair of DNA interstrand crosslinks (ICL) during DNA replication [5]. A key event in FA pathway activation is the monoubiquitination of FANCI-FANCD2 (ID) heterodimers by the FA “core complex” (FANCA/B/C/E/F/G/L/M) [6]–[8]. The monoubiquitinated ID complex is recruited to DNA ICLs, and coordinates ICL repair together with downstream FA proteins (D1/J/N/O/P) and other (BRCA1, ATR) DNA repair proteins [1], [9], [10]. FANCM complexed with FAAP24 initiates FA pathway activity by recognizing DNA damage and loading the FA core complex. FAAP24 is particularly important in activating ATR in response to ICLs [11]. FANCM also has translocase activity that promotes branch migration of Holliday junctions and replication forks independent of FAAP24 [12].

FA deficient cells are hypersensitive to agents that induce ICLs, such as mitomycin C [MMC] or cisplatin. Most FA patients manifest anemia and bone marrow failure during childhood and are predisposed to cancer. Reduced fertility, hypogonadism and testicular failure, which is a consequence of impaired gametogenesis, are also common [13], [14], and this is reflected in most mouse models for FA, including knockouts for *Fanca*, *Fancc*, *Fancd2*, *Fancf*, *Fancg*, *Fancl*, *Fancm*, and *Fancp*, though *Fancd1* is an exception [15]–[22]. While the severity varies amongst mutants, males generally present a partial Sertoli Cell Only-like phenotype whereby a subset of seminiferous tubule sections are depleted of germ cells. In mutant females, the number of ovarian follicles is typically reduced. Although most of these mutants have been characterized only as adults, the germ cell defects in three have been investigated perinatally or earlier. Germ cell depletion in *Fancd2* ^−/−^ is evident in newborn mice [22], and defects in the proliferation of PGCs were reported in *Fancc* and *Fancl* mutants [15], [23]. While defects in DNA repair presumably underlie these germ cell phenotypes, the downstream DNA damage signaling pathway(s) that respond to these defects, ultimately leading to germ cell depletion, have not been identified.

The FA pathway appears to function in all cell types, including germ cells. However, experimental difficulties in studying the mammalian germline – particularly those stages occurring during embryonic development – have limited investigations into the roles of the FA and other DNA damage response (DDR) pathways in these cells. Importantly, the germline mutation rate is significant lower than that in somatic cells [24], [25], indicating a fundamental difference in genome maintenance that appears to reflect the biological importance of minimizing the germline mutation rate. While specific DDRs in the *C. elegans* germline have been identified [26], the DDRs operative in mammalian PGCs have not.

Here we investigate a *Fancm* mouse model (*Fancm^Chaos4^*) that was recovered in a forward genetic screen for GIN mutants. Mutant mice exhibit GIN and PGC depletion during embryogenesis. Using a genetic approach, we found that the ATM-p53-p21 axis contributes to the PGC depletion in this model, underscoring the critical importance of genome maintenance in these cells that undergo rapid cellular proliferation during a short period of time during development.

## Results

### Isolation of a new *Fancm* allele, *Fancm^Chaos4^*, from a forward genetic screen for GIN mutations in mice

**Figure 1:**
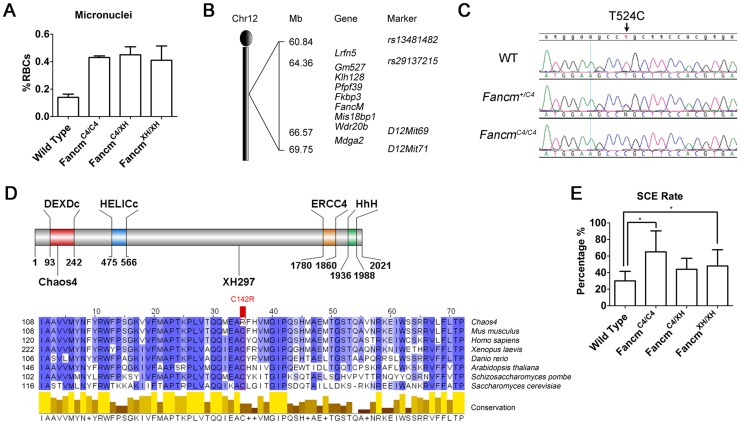
The *Chaos4* allele is a point mutation in *Fancm*. (A) Flow cytometric analysis of erythrocytes to quantify red blood cells (RBC) with micronuclei. (B) Genetic mapping of *Chaos4* to a 9-Mb region of chromosome 12 containing *Fancm* between *rs13481482* and *D12Mit71*. (C). Sequence traces showing the T524C transversion (arrows) identified in the *Chaos4* allele of *Fancm*. (D) The *Chaos4* point mutation is in the first exon, and the *XH297* gene-trap is in the 14th exon. DEXDc, DEAD-like helicase domain; HELICc, Helicase superfamily c-terminal domain; ERCC4, ERCC4 endonuclease domain; HhH, Helix-hairpin-helix domain which interacts with FAAP24. Sequence alignment surrounding C142 is highly conserved from human to budding yeast. (E) SCE rates are significantly increased in *Fancm^C4/C4^* MEFs (p<0.05).

We previously conducted an *N*-ethyl-*N*-nitrosourea (ENU) mutagenesis screen in mice for mutants showing chromosome instability, as assessed by micronucleus levels in erythrocytes [27]. *Chaos4* (chromosome aberrations occurring spontaneously 4) was one mutation identified in this screen. Homozygous mutants show a mildly elevated (3 fold) frequency of erythrocytes with micronuclei (Figure 1A). Using combined SNP- [28] and microsatellite-based mapping, *Chaos4* was genetically localized to a 9-Mb region between *RS13481482* and *D12Mit71* containing 9 RefSeq genes, including *Fancm* (Figure 1B). Sequencing of *Fancm* cDNA from mutants and controls identified a *de novo* T to C transition at nucleotide 524 of the coding region (Figure 1C). This point mutation changes a highly conserved cysteine residue to arginine (C142A) that is located within the DEXDc domain of this DEAD-like helicase superfamily region of FANCM (Figure 1D).

To confirm that the point mutation in *Chaos4* underlies the GIN phenotype, we performed complementation analysis with a *Fancm* gene-trap allele, *Fancm^Gt(XH297)Byg^*, abbreviated hereafter as *Fancm^XH^*. The gene-trap vector resides in exon 14, between the helicase and endonuclease domains (Figure 1D). *Fancm^XH^* homozygotes also had elevated erythrocyte micronuclei (Figure 1A) as did *Fancm^C4/XH^* mice, providing strong evidence that the *Fancm^Chaos4^* allele (hereafter abbreviated *Fancm^C4^*) is responsible for the GIN phenotype. We further assessed the chromosomal instability phenotype of our alleles via the sister chromatid exchange (SCE) assay. Consistent with results from a *Fancm^Δ2^* knockout mouse model [18], untreated *Fancm^C4/C4^* and *Fancm^XH/XH^* MEFs both had elevated DNA breaks and radial chromosomes (Figure 1E; Figure S1), further confirming that the Chaos4 phenotype is attributable to the mutation in *Fancm*. Both *Fancm^C4/C4^* and *Fancm^XH/XH^* mice were born at a Mendelian ratios, indicating that the mutations do not compromise embryonic viability (Table S1).

### *Fancm^C4/C4^* primary MEFs undergo premature immortalization and mutant mice are cancer prone

**Figure 2:**
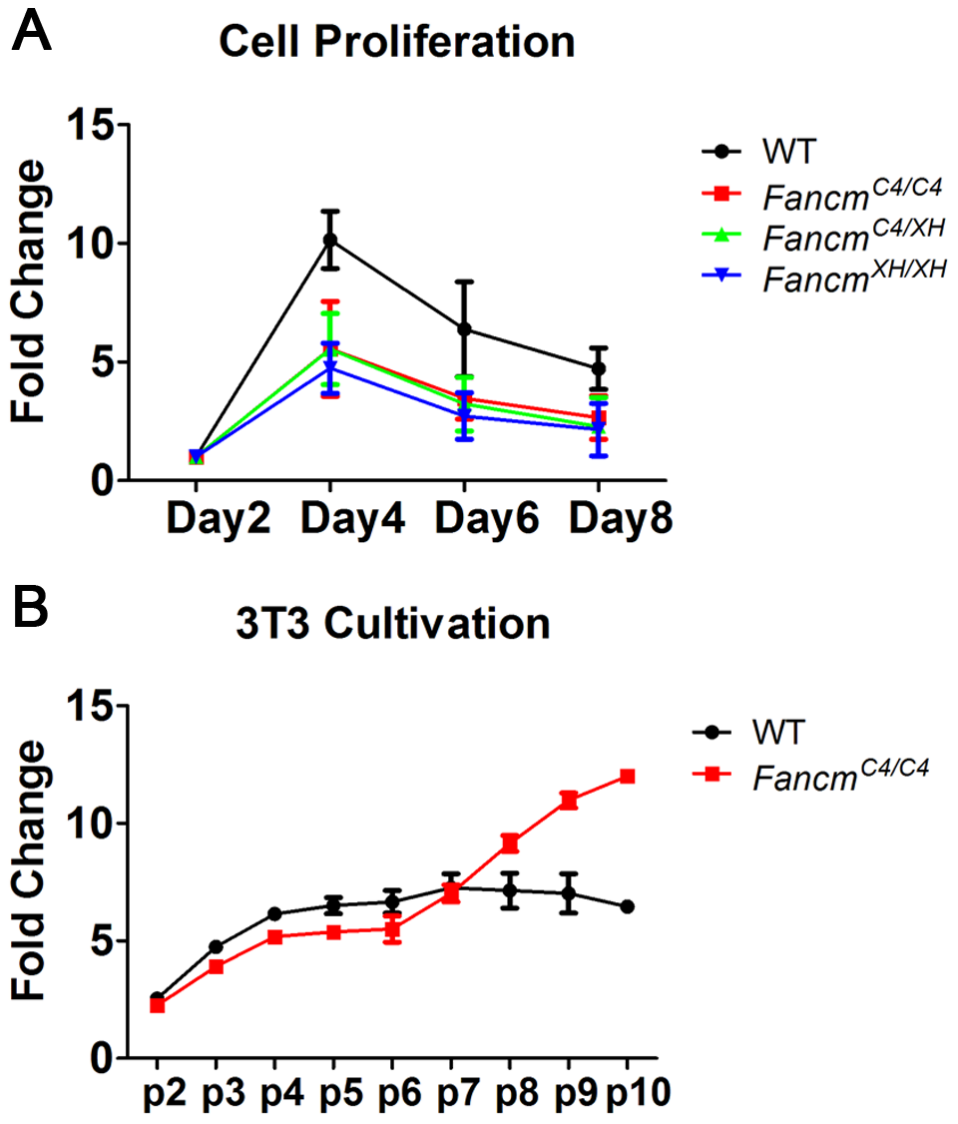
*Fancm^C4/C4^* MEFs undergo premature senescence, but are not sensitive to interstrand crosslinks. (A) MEF growth assays. (B) Immortalization timeline of primary MEFs using a 3T3 growth protocol. Cultures were passaged every 3 days.

The proliferation of untreated *Fancm^C4/C4^* primary MEFs during early passages was diminished compared to wild-type (Figure 2A, B). However, they recovered from senescent crisis and became immortalized much earlier (by passage 7) than wild-type (passage 10 or later) (Figure 2A, B).

Cancer predisposition is a defining feature of Fanconi Anemia. To determine if the early immortalization was an indicator of cancer susceptibility, *Fancm^C4^* mutants were aged for up to 1.5 years. *Fancm^+/C4^* and *Fancm^C4/C4^* females congenic in the C3HeB/FeJ background had significantly elevated cancer/neoplasia susceptibility (Table S2), developing multiple tumor types (Table S3). Thirty-three percent (33%) of heterozygotes (9/27) and 58% of homozygous females (15/26) developed tumors by ∼1 year of age, compared to none of the 28 WT controls (p = 0.004 and p = 0.0002, respectively). The most common tumor types were ovarian, mammary and uterine. Heterozygous and homozygous *Fancm^C4^* males also were significantly tumor prone (42%, p = 0.001 and 47%, p = 0.002, respectively, vs. 9% of WT males; Tables S2, S3). *Fancm* null mice were reported to have a similar degree of tumor susceptibility [18].

### FANCM deficiency compromises primordial germ cell proliferation and causes meiotic defects

**Figure 3:**
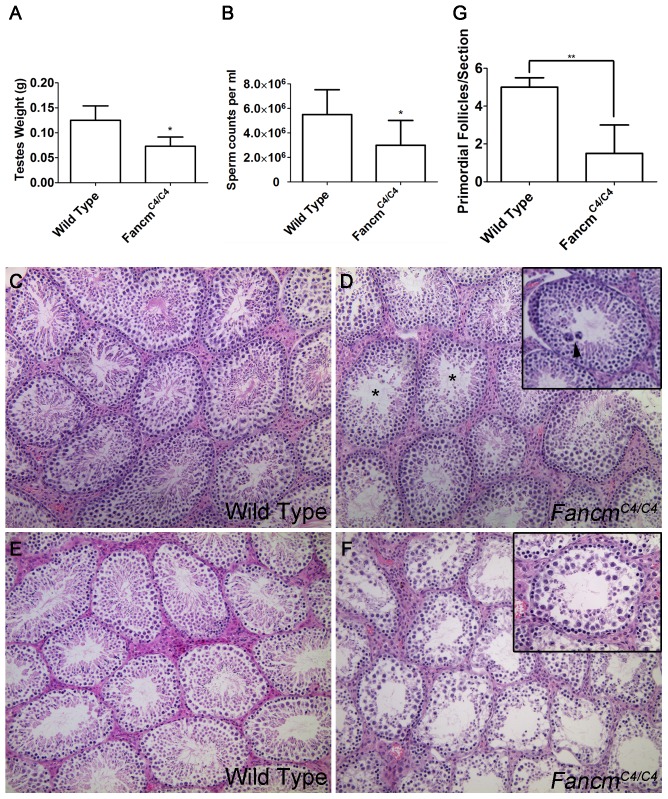
Hypogonadism and spermatogenesis defects in *Fancm* mutant males. (A,B) *Fancm^C4/C4^* mice (12 weeks old) have smaller testes and lower epididymal sperm counts. (C–F) H&E-staining of testis sections of the indicated genotypes. Samples in panels C–D are from 16 week old males. Arrowhead points to a giant multinucleated cell. (*) indicates an example of a seminiferous tubule with abnormal spermatogenesis. Panels E and F are from 80-wk-old males. Most tubules in 80 week *Fancm^C4/C4^* testes have only Sertoli cells. (G) Primordial follicles quantification in mutants. * p<0.05, ** p<0.01 n>10 for each genotype.

In a limited gross and histological study, adult *Fancm* null mice were reported to have smaller gonads, germ cell loss in a subset of seminiferous tubule sections, and a reduced number of ovarian follicles [18]. Similar to those findings, we found that although *Fancm^C4/C4^* males appear grossly normal and were fertile, they had markedly smaller testes and about 60% the amount of sperm as wild-type littermates at 12 weeks of age (Figure 3A,B). Testis histology of young mice (≤16 weeks of age) revealed subtle seminiferous tubule abnormalities, namely the presence of occasional giant multinucleated cells that are not present in WT (Figure 3C, D). Prior to inbreeding onto strain C3HeB/FeJ, young *Fancm^C4/C4^* also exhibited germ-cell depleted individual tubules (not shown). Spermatogenesis defects in *Fancm^C4/C4^* mice (but not WT controls) became more severe over time, such that most seminiferous tubules in mice over 1 year of age were highly disrupted (Figure 3E, F). Gonadal defects in *Fancm^C4/C4^* mutants were sex independent; females manifested a significant depletion of primordial follicles compared to WT animals (Figure 3G).

**Figure 4:**
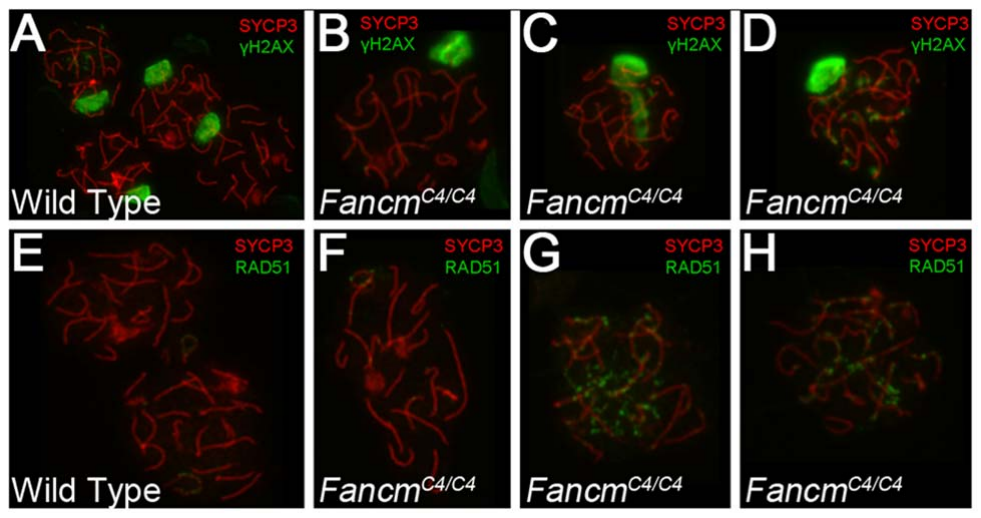
Meiotic defects in *Chaos4* mutant spermatocytes. (A–H) Shown are surface spread meiotic chromosomes from the indicated genotypes of males. Antibodies used for immunolabeling are as indicated with color coding. SYCP3 is a synaptonemal complex protein marking chromosome cores. The large domains of γH2AX staining in A,B and D correspond to the XY body. In panel C, there is an extended region of XY body-like staining over autosomes, a pattern typically called a “pseudo sex body” and usually marks asynapsed autosomes. All nuclei are in the pachytene stage of meiosis.

The presence of multinucleate cells in younger animals was suggestive of abnormal meiotic or premeiotic cell divisions. To investigate potential meiosis defects, we immunolabeled meiotic chromosomes from 12-week *Fancm^C4/C4^* males with markers of DSB signaling (γH2AX, the phosphorylated form of H2AX), DSB repair (RAD51), and meiotic chromosome structure (SYCP3, which detects axial elements of the synaptonemal complex). H2AX phosphorylation is also a marker of, and is involved in, transcriptional Meiotic Silencing of Unsynapsed Chromatin (MSUC) during meiosis [29]. As in WT (Figure 4A, E), most mutant pachytene spermatocytes had a normal XY body (marked by an intense γH2AX domain) and no RAD51 foci or autosomal γH2AX staining (Figure 4B, F), indicative of proper chromosome synapsis and recombinational repair of programmed (SPO11-induced) meiotic DSBs. However, 42% of the pachytene nuclei showed abnormal γH2AX staining, either spreading as a cloud into autosomes (Figure 4C) or as punctate foci on chromosome axes (Figure 4D), reflective of unsynapsed chromosomes and unrepaired DSBs, respectively. Consistent with the γH2AX results, twenty-seven percent of the spreads showed persistent RAD51 foci (Figure 4G, H). The data suggest that *Fancm^C4/C4^* spermatocytes have a defect in meiotic DSB repair, which in turn may affect synapsis of chromosomes in a subset of spermatocytes.

**Figure 5:**
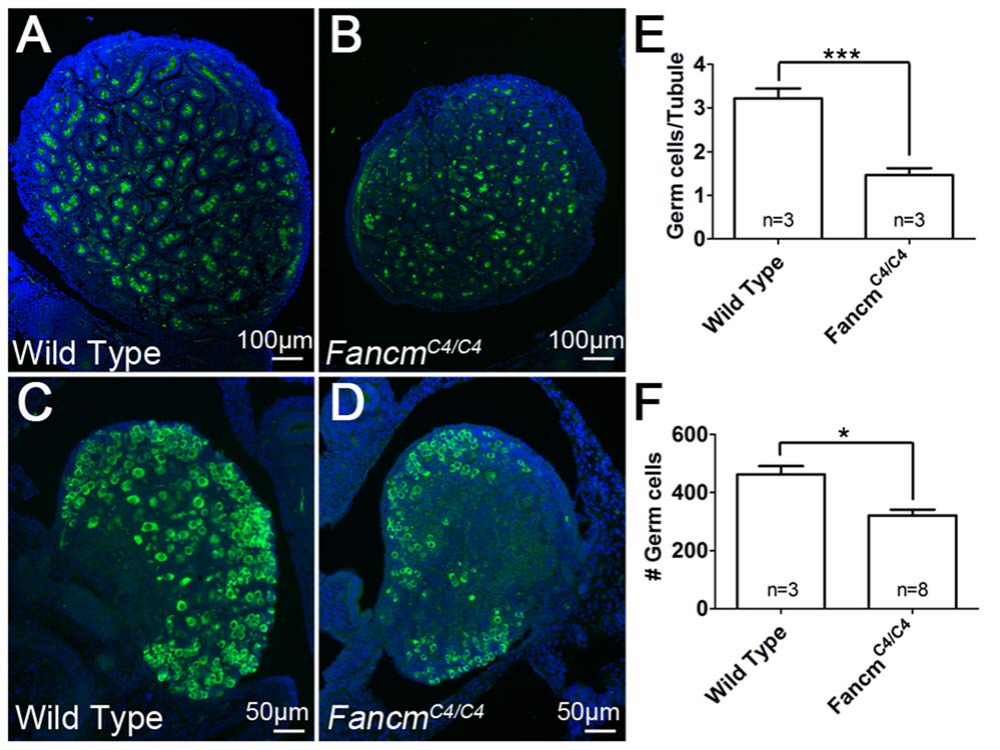
Germ cell depletion in *Fancm^C4/C4^* occurs before birth. Immunofluorescence of 1(A and B) and ovaries (C and D) of the indicated genotypes. *MVH* (green) stains germ cells and DAPI stains nuclei (blue). (E and F) Germ-cell counts at 1 dpp. Germ cell number is averaged on a per seminiferous tubule cross-section basis for males. Female counts correspond to the total from three medial sections. **, p<0.01; ***, p<0.001; Error bars indicate SD.

The incomplete, sex-independent germ cell depletion in young adults, characterized by primordial follicle reduction, reduced testis size, and germ cell losses in some seminiferous tubules was suggestive of premeiotic germ cell defects. To explore this, newborn gonads were serially sectioned and probed with the germ cell-specific marker MVH (mouse vasa homolog) to quantify the number of germ cells at birth. In *Fancm^C4/C4^* males and females, there were markedly fewer germ cells (55% and 30%, respectively) compared to wild-type littermates (Figure 5). This indicates that the germ cell depletion is initiated during embryogenesis.

**Figure 6:**
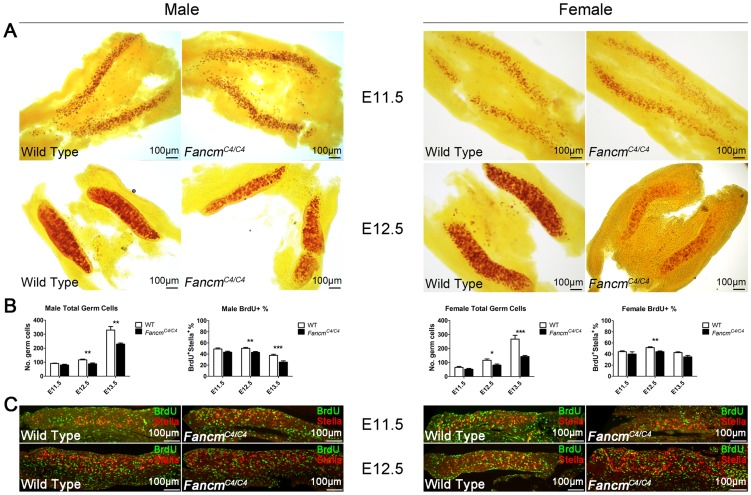
PGC depletion in *Fancm^C4/C4^* mice is associated with reduced proliferation, not apoptosis. (A) Male and female embryonic gonads from E11.5 and E12.5 stained for alkaline phosphatase activity. A decrease in PGCs is becomes evident only at the latter time point. The graphs of germ cells in (B) represent quantification of germ cells by immunolabelling fetal gonads with either Stella (E11.5 and E12.5) or MVH (E13.5). Representative images for E13.5 gonads are shown in Figure S2. The graphs of BrdU+ cells represent data from BrdU incorporation assays shown in (C and Figure S2). BrdU and Stella double-positive cells, which represent PGCs in S phase, were quantified as percentage of total Stella-positive PGCs.

To identify the stage at which germ cell depletion starts, we examined the PGC population at various times of gestation. PGCs are first specified extra-embryonically at embryonic day 7.5 (E7.5). Between E8.5 and E10.5, this pool of alkaline phosphatase-positive PGCs then migrates along the epithelia of the hindgut towards the urogenital ridge, undergoing a modest degree of proliferation along the way. From there, they traverse the dorsal mesentery and populate the primitive gonad. They then undergo a dramatic proliferation after which male PGCs enter mitotic arrest until 3–4 dpp, while female PGCs enter meiosis at ∼E13.5 and arrest in meiotic prophase I until puberty (reviewed in [30]). We quantified PGCs at E11.5, E12.5 and E13.5. The numbers were not significantly decreased in either male or female *Fancm^C4/C4^* embryos at E11.5 (Figure 6). However, a significant reduction was evident by E12.5 and E13.5 (Figure 6).

These combined data suggest that FANCM deficiency does not significantly impair PGC specification or migration, but rather that mutant PGCs either proliferate more slowly or undergo elevated apoptosis. To distinguish between these possibilities, we assessed PGC proliferation and apoptosis using BrdU incorporation and TUNEL assays, respectively. The BrdU incorporation assays indicated that PGC proliferation is reduced in both male and female *Fancm^C4/C4^* gonads at E12.5 and E13.5 (Figure 6B; Figure S2). Furthermore, apoptosis was not evident in either wild type or *Fancm^C4/C4^* gonads at E12.5 (Figure S3).

Previous studies estimated the number and the doubling time of PGCs between E11.5 and E13.5 [31], [32]. The doubling time of wild type PGCs is 15.8 h in males, and 16.1 h in females (see Methods). Based on our PGC quantification, the doubling time of *Fancm^C4/C4^* PGC increased to 17.9±0.2 h in males, and 18.9±0.3 h in females.

### DNA damage response pathways involved in PGC depletion

Although hypogonadism and testicular failure is characteristic of FA, a possible link between this and FA-related GIN has not been established. We hypothesized that if activation of a particular DDR pathway triggers PGC growth arrest or attenuation, then genetic disruption of that pathway would relieve the PGC depletion. Accordingly, we crossed *Fancm^C4/C4^* with various checkpoint mutants, including alleles of *Atm*, *Chk2* (*Chek2*), *p53* (*Trp53*), *p21* (*Cdkn1a*), and *Hus1* to obtain double mutants. All mutations were congenic or near congenic (at least 7 backcross generations) on the C3H strain background. The numbers of MVH-positive germ cells in newborn gonads were then quantified.

**Figure 7:**
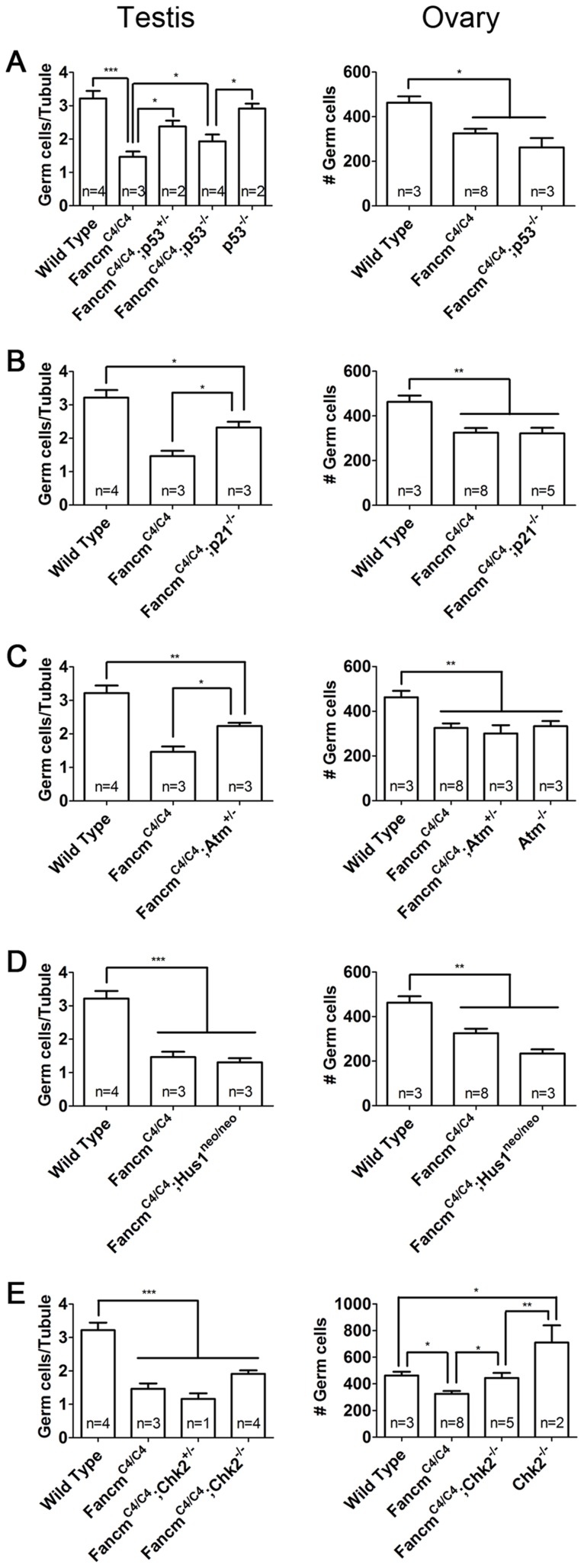
Genetic analysis of checkpoint signaling in *Fancm^C4/C4^* germ cells. (A–E) Compound mutant gonads with indicated genotypes were collected at 1 dpp. Germ-cell counts were performed following MVH labeling. Male germ cell number was averaged on a per tubule cross-section basis for males. Values for females equal the total of germ cells detected in three medial sections. *, p<0.05; **, p<0.01; ***, p<0.001; Error bars indicate SD.

We first analyzed the role of p53 and its downstream effector p21 [33], [34]. Deletion of one or both p53 alleles partially but significantly rescued germ cell loss in *Fancm^C4/C4^* male newborns (Figure 7A). This partial rescue implies that some but not all germ cell depletion is due to p53 activation. Similar partial rescue was observed in *Fancm^C4/C4^ p21^−/−^* males (Figure 7B). The involvement of p21, a CDK inhibitor and downstream effector of p53 [35], [36], is consistent with our previous finding that PGC depletion in *Fancm^C4/C4^* is a result of reduced proliferation. Surprisingly, the partial rescue was sexually dimorphic; neither *p53* nor *p21* knockout ameliorated the germ cell deficiency in newborn *Fancm^C4/C4^* females.

Next, we focused on the upstream kinases of two major DDR pathways, ATM and ATR [37]. These two proteins primarily respond to DSBs and sites of replication errors (RPA-coated ssDNA), respectively. Intercrosses of *Fancm^C4/C4^ Atm^+/−^* mice produced 49 pups, none of which were homozygous for both mutations (p<0.001; expected = 12.25). Whereas doubly deficient mice were not born, *Fancm^C4/C4^* mice heterozygous for *Atm* were viable, and the genetic reduction of ATM partially rescued the germ cell loss in males but not females (Figure 7C). Therefore, *Atm* may respond to increased DNA damage in *Fancm^C4/C4^* PGCs, ultimately activating p53-p21 signaling to protect the fidelity of genetic information in the PGC pool. In contrast, a hypomorphic viable allele (*Hus1^neo^*) of the ATR-pathway gene *Hus1* [38] had no apparent impact on the depletion of *Fancm^C4/C4^* PGCs (Figure 7D).

Given the partial phenotypic rescue of *Fancm^C4/C4^* PGCs by *Atm* haploinsufficiency and *p53* nullizygosity, we hypothesized that the ATM target CHK2 served as the intermediate transducer kinase. However, *Chk2* deficiency did not rescue germ cells loss in *Fancm^C4/C4^* males, but significantly rescued the germ cell population in *Fancm^C4/C4^* females (Figure 7E). Interestingly, *Chk2^−/−^* newborn females had more germ cells than WT controls (Figure 7E). Therefore, the rescue effect of *Chk2* mutation is probably independent of *Fancm^C4^* mutation. As previously reported [39], we observed that *Chk2^−/−^* adults had histologically normal gonads. *Chk2^−/−^* males did not have more gonocytes at birth than WT siblings (Figure 7E). Since female but not male PGCs enter meiosis before birth, and *Chk2* was recently found to play a crucial DNA damage checkpoint role in female meiosis [40], this may account for the elevated number of oocytes in double mutants.

## Discussion

FANCM is a key component of the FA signaling pathway. Numerous *in vitro* studies have suggested that FANCM is a sensor of DNA damage at replication forks and helps anchor the FA core complex to chromatin [8], [41]–[44]. *Fancm* was also reported to have the non-canonical function of regulating meiotic crossovers in *Arabidopsis thaliana* and *Saccharomyces pombe*, specifically by catalyzing interference-independent recombination intermediates to undergo noncrossover rather than crossover resolution [45]–[47]. It was recently shown that FANCM, via its translocase activity, interacts with MHF to allow replication to “traverse” ICLs without repair, and that this activity is independent of other FA members [48]. Despite the substantial biochemical and mechanistic information on *Fancm* function, the physiological roles of *Fancm* in vertebrates are incompletely characterized.

A previous study found that *Fancm* null mice not only phenocopied other FA mouse models in causing hypogonadism and hypersensitivity to cross-linking agents (in MEFs), but also had decreased longevity and tumor-free survival [18]. As with the null mutant, *Fancm^C4/C4^* mice had elevated SCE and tumor susceptibility, and *Fancm^C4/C4^* MEFs underwent senescence prematurely. The general similarity in phenotypes between the null and *Fancm^C4^* alleles indicates that the single amino acid change in the DEAH helicase domain disrupts the crucial function of this protein in mice. This domain has no detectable helicase activity, but does encode the translocase activity of FANCM that is important for promoting the recovery of stalled replication forks [49], [50]. Given that mutating the translocase function of FANCM alone disrupts replication traverse of ICLs in the same manner as null cells [48], we speculate that the *Fancm^C4^* mutation disrupts translocase function to yield phenotypes that are essentially indistinguishable from nulls. Future studies to test this and other possibilities, such as protein stability, would be of interest.

We traced the cause of germ cell depletion in newborn FANCM-deficient mice to defects in PGC proliferation, which was not reported for the knockout, but which has been noted for knockouts of other FA genes (discussed earlier). Specifically, we found that the ATM-p53-p21 DDR pathway is operative in regulating PGC proliferation in males. Mutations of each partially restored germ cell numbers in newborns. However, the results with compound *Atm* mutants suggest a complex relationship with FANCM in PGCs. It has been reported that FANCM is actually regulated in part by ATR and ATM in response to damaged DNA in a *Xenopus* extract system [51], but the synthetic lethality between *Fancm* and complete ATM deficiency (*Atm^−/−^*) suggests that ATM and FANCM also have parallel, non-epistatic roles in DDRs during development. The *Fancg^−/−^ Atm^−/−^* genotype also causes embryonic lethality [52], and inhibition of the FA pathway selectively kills ATM-deficient cells [53], [54], supporting the idea that the DNA damage to which the ATM and the FA pathway responds overlap. The viability of, and partial rescue of PGC loss in, *Fancm^C4/C4^ Atm^+/−^* males suggests that the parallel DNA repair role of reduced ATM is sufficient to overcome the lack of functional FA pathway repair, but compromises checkpoint-mediated cell cycle delay in PGCs, presumably via reduced signaling to p53.

**Figure 8:**
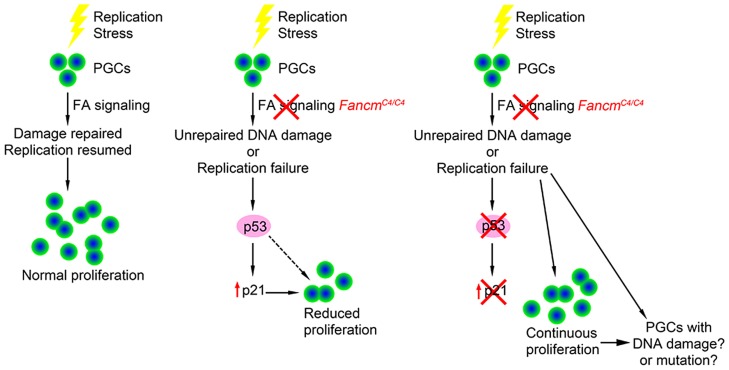
Model of checkpoint responses to replication stress in primordial germ cells.

p53 is a key transcription factor that regulates several signaling pathways involved in the response to cellular stress, DNA damage, oncogene activation and other physiological signals [55]. Genetic experiments in mice have shown that p53 plays a role in FA signaling. p53 deficiency partially rescues the embryonic lethality in *Fancn* (*Palb2*) and *Fanco* (*Rad51c*) mutants [56], [57] and bone marrow failure in *Fancd2* mutants [58]. Our studies provide the first evidence that p53 is involved in genome surveillance of PGCs during their expansion phase in development, at least in males. In the context of *Fancm* deficiency and the presumed increase of DNA lesions this causes, p53 appears to slow cell cycle progression rather than causing apoptosis (see model in Figure 8). Mutations in *Fancl* and *Fancc* also cause germ cell reduction traced to reduced PGC proliferation and not apoptosis [15], [23], suggesting that the level of endogenous DNA damage induced by FA pathway defects is not sufficient to stimulate p53-mediated apoptotic signaling. In contrast, p53 was reported to mediate germ cell apoptosis in Zebrafish *fancl* mutants [59], implying either that germ cells in this organism are more sensitive to DNA replication defects, the p53 pathway is more active in zebrafish germ cells, and/or zebrafish lack a redundant repair pathway(s).

The activity of p53 alone doesn't fully account for germ cell depletion in *Fancm* mutants. Aside from only partial rescue in *Fancm^C4/C4^* males by *p53* deletion, which suggests that an additional or parallel DDR pathway might still be operative such as one involving paralogs p63 and p73, p53 deficiency did not rescue loss of oocytes in newborn females. One possible explanation for this sexual dimorphism may relate to the direct entry of female PGCs into meiosis at ∼E13.5, unlike the mitotic arrest that male PGCs undergo. Since quantification of germ cell number in compound mutants was conducted in newborns, the number of oocytes at birth reflects events that occur both during PGC proliferation and during meiotic prophase I. Considering that male *Fancm^C4/C4^* meiocytes had substantially elevated DSBs, and mouse oocytes have a stringent meiotic DNA damage checkpoint that causes apoptotic elimination perinatally [60], it is possible that any rescue of PGC proliferation in *Fancm^C4/C4^ p53^−/−^* females was counteracted by subsequent meiotic losses of those oocytes derived from damage-bearing “rescued” PGCs. Importantly, the oocyte DNA damage checkpoint involves signaling of CHK2 to both p53 and TAp63, and that in the absence of p53, DSB-bearing oocytes are still efficiently eliminated by CHK2-activated TAp63 [40]. As mentioned earlier, our observation that perinatal *Fancm^C4/C4^* germ cell numbers were rescued in CHK2-deficient females but not males likely reflects this oocyte-specific meiotic DNA damage pathway, not a PGC DDR.

Few DNA repair gene mutations are known to impact PGC growth or maintenance. Beyond FA mutants, *Pin1*, *Mcm9*, *Rev7* and *Helq* are four other genes that have been correlated with both a function in genome maintenance and a PGC depletion phenotype [61]–[66]. *Pin1* is a prolyl isomerase which directly regulates cell cycle genes. *Pin1* deletion depletes PGCs by delaying their proliferation [64]. *Mcm9* and *Helq* appear to be involved in homologous recombination repair (HRR) of ICLs. HELQ interacts with the RAD51 paralog complex, but appears to function in a pathway in parallel to FA [61], [62], [67]–[70]. MCM9 is required for normal homologous recombination, promoting recruitment of RAD51 to DNA damage sites and repair of ICLs [68]–[70] It also appears to act downstream of the FA pathway [70]. Interestingly, FANCM was reported to be required for HR-independent ICL repair [11]. Despite these indications of multiple pathways for DNA repair in PGCs, that these cells remain highly sensitive to perturbations of any of them.

*Fancm^C4/C4^* males also exhibited progressive germ cell depletion with age. The reason for this is unclear, since histological analysis revealed only subtle seminiferous tubule abnormalities in young mice. The progression to a near Sertoli Cell Only-like phenotype in many tubules suggests a defect in spermatogonial proliferation or renewal. The lack of more dramatic testicular pathology in young mice is also curious in light of evidence for DNA repair and XY-body defects in a substantial fraction of spermatocytes. Aside from the occasional appearance of abnormal multinucleated cells near the lumen of seminiferous tubules, coordinated arrest of pachytene stage spermatocytes was not observed as is typical for mutants that are recombination-defective and which disrupt XY silencing, an event proposed to underlie meiotic arrest [71]. One possible explanation is that the level of defects is below the threshold that would trigger a checkpoint, or that the unrepaired DNA damage is eventually repaired before checkpoint-mediated elimination. It may be relevant in this regard that we have not noticed visual abnormalities in offspring of *Fancm* mutants. Another possibility is that the DNA damage in *Fancm^C4/C4^* spermatocytes, inferred as such by the presence of γH2AX and RAD51 foci, may be of a nature that does not trigger elimination. For example, it is possible that these foci correspond to sites of damage incurred during premeiotic DNA replication, as opposed to SPO11-induced DSBs. Another example of apparently tolerated meiotic damage is the case of *Rad54^−/−^* spermatocytes, which are not eliminated despite bearing extensive RAD51 foci in late pachynema [72]. Finally, it is possible that *Fancm* has a hitherto unknown role in meiotic checkpoint activation in addition to DNA repair.

This study contributes to an emerging picture that the FA pathway is particularly important in stem cell biology [2]. Reprogramming of fibroblasts into induced pluripotent stem cells requires FA pathway function [73], [74]. Furthermore, not only is bone marrow failure a hallmark of FA, but this failure depends upon p53/p21 signaling [58]. The involvement of p53/p21 activation in both hematopoietic and germline stem cells bearing FA mutations, and the particular sensitivity of these lineage, emphasizes the importance of expanding studies of the FA pathway into diverse cell types including additional stem cell lineages.

## Materials and Methods

### Micronucleus assays

These were performed as described [75].

### Positional cloning

The *Chaos4* mutation was ENU-induced on the C57BL/6J (“B6”) background [27]. To identify the causative mutation, the mutation was outcrossed to strain C3HeB/FeJ (“C3H”), then intercrossed to produce potential homozygotes. F2 offspring were screened for micronucleus levels and a genome scan with a collection of microsatellite markers polymorphic between C3H and the parental strain B6 was performed [28]. This localized *Chaos4* to a 44-Mb interval on chromosome 12, between *D12Mit285* and *D12Mit71*. Subsequently, we conducted an inter-subspecific mapping cross with *Mus castaneus* (CAST/Ei). The F1s were either intercrossed or backcrossed to CAST/Ei and scored for micronuclei. A total of 956 informative meioses were examined, defining a 9-Mb critical region (Figure 1B).

### Embryonic stem cell culture and microinjection for chimera production

The XH297 ES cell line (derived from the 129/Ola strain; BayGenomics) [76] bearing a gene trap insertion of *Fancm* (abbreviated *Fancm^XH^*) were cultured in DMEM (Gibco) supplemented with 15% FBS (HyClone), 0.1 mM MEM nonessential amino acids, 1 mM sodium pyruvate, penicillin-streptomycin (100 units/ml), 100 µM beta-mercaptoethanol (Sigma) and recombinant leukemia inhibitory factor (produced in-house). Cells were microinjected into C57BL/6J blastocysts by standard methods. *Fancm^+/XH^* mice were then backcrossed into the C3HeB/FeJ background.

### Mice and genotyping

Genotyping of *Fancm^C4^* mice was performed by PCR amplification of a 240 bp mutated segment with two primers: Chaos4L (CTTCTGGCAAGGTGGTTTTC) and Chaos4R (TTTGCTACCCACAGACGATG). PCR products were then digested by restriction enzyme *Aci*I, which is present in the *Chaos4* allele only. The *Chaos4* allele is cut into 180 bp and 60 bp fragments. Genotyping of *Fancm^XH^* mice was performed indirectly using microsatellite markers *D12Mit69* and *D12Mit71* that flank *Fancm*, and which are polymorphic between strain C3H and B6 (B6 alleles at *D12Mit69* and *D12Mit71* are indicative of the *Chaos4* allele). The use of mice in this study was approved by Cornell's Institutional Animal Care and Use Committee. Mice bearing alleles of other mutations were: *Atm (Atm^tm1Led^, abbreviated as Atm^−^)*, *Chk2 (Chek2^tm1Mak^, abbreviated as Chk2^−^)*, *p53 (Trp53^tm1Tyj^, abbreviated as p53^−^)*, *p21 (Cdkn1a^tm1Tyj^, abbreviated as p21^−^)*, and *Hus1 (Hus1^tm2Rsw^, abbreviated as Hus1^neo^)* [39], [77]–[80]. The stocks of mice bearing the *p53*, *p21* and *Hus1* alleles were all congenic in the C3H background (N10 or greater). The *Atm*, *Chk2* stocks were at the N7 backcross generation. Euthanasia was performed by CO_2_ administration.

### Mouse embryonic fibroblast (MEF) growth analyses

MEFs were generated from 12.5- to 14.5-dpc embryos. Cells were cultured in DMEM supplemented with 15% FBS (fetal bovine serum), 0.1 mM MEM nonessential amino acids, 1 mM sodium pyruvate, penicillin-streptomycin (100 units/ml), and beta-mercaptoethanol. For cell proliferation assays, 0.5×10^6^ cells were seeded per 100-mm plate and then cultured and harvested to count cell numbers at various time points. For the cell senescence assay, 0.5×10^6^ cells were seeded per 100-mm plate and then cultured and passaged every 3 days until they became immortalized. MEF metaphase spreads and the sister chromatid exchange assay were performed as previously described [18], [81].

### Histology and immunohistochemistry

For basic histology, tissues were fixed in 4% paraformaldehyde (PFA) overnight, paraffin-embedded, sectioned at 5 µm, and stained with H&E (hematoxylin and eosin). Statistical differences in tumor types were assessed via Fisher's exact test. For germ-cell counts on embryonic or newborn gonads, 5 µm sections were immunostained as previously described [82]. Germ cells in postnatal gonads were counted in three sections from the midportion of each gonad and averaged. Antibodies: Rabbit anti-DDX4/MVH (Abcam ab13840; 1∶250); rabbit anti-Stella (Abcam ab19878; 1∶250); goat anti-mouse Alexa 594 conjugate (Molecular Probes A11005; 1∶1,000); goat anti-rabbit Alexa 488 conjugate (Molecular Probes A11008; 1∶1,000). The data were analyzed using one-way ANOVA with Bonferroni correction (Prism software package). The resulting P values were used to determine significance (P<0.05).

### BrdU incorporation assay

Pregnant females received a single BrdU intraperitoneal injection (50 mg/kg) at 11, 12, or 13 days after vaginal plug detection (their corresponding embryos were E11.5, E12.5 and E13.5). Injected mice were sacrificed two hours later, and embryos were collected. Embryonic gonads together with mesonephric tubules (for E12.5 and E13.5 embryos) or the dorsal part of the trunk without other internal organs (for E11.5 embryos) were fixed in 4% PFA. Tissues were embedded in paraffin and sectioned. BrdU was detected by the Invitrogen BrdU Staining Kit (Cat. No. 93-3944), and PGCs were detected with rabbit anti-Stella (Abcam ab19878; 1∶250). At least three sagittal sections across the central part of the gonads were used for PGC quantification and BrdU scoring.

Since no cell apoptosis was obvious and no cell migration occurs between E11.5 and E13.5, PGC doubling time was calculated based on an exponential growth model:
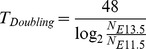
 N*_E13.5_* and N*_E11.5_* are the absolute number of PGCs in the whole gonad, which was estimated based on the previous studies and the relative ratio between wild type and mutants.

### Alkaline phosphatase staining

Embryonic gonads were stained as described [83]. Briefly, fixed gonads were washed with dH_2_O and stained with freshly made staining solution (0.1 mg/ml Sodium α-naphthyl phosphate, 5 mg/ml Borax, 0.6 mg/ml MgCl_2_, and 0.5 mg/ml Fast Red TR salt) for 15–30 min. Tissues were then washed in dH_2_O and cleared with 70% glycerol.

### TUNEL staining

Five µm paraffin sections of embryonic gonads were TUNEL stained using the In Situ Cell Death Detection Kit (Roche 11684817910). *Atm^−/−^* adult testes were used as a positive control [84].

### Meiotic chromosome analysis

This was performed as described [75]. Primary antibodies used in this study: rabbit anti-SYCP3 (1∶500, Abcam); mouse anti-γH2AX (1∶500, JBW301 Upstate Biotechnology); rabbit anti-RAD51 (1∶250, this polyclonal antibody recognizes both RAD51 and DMC1; Oncogene Research Products).

### Ethics statement regarding vertebrate animal use

The use of mice in this study was approved by Cornell's Institutional Animal Care and Use Committee, under the approved protocol of JCS (2004-0038). Euthanasia was performed by CO_2_ administration.

## Supporting Information

Figure S1 Chromosomal instability in *Fancm* mutant MEFs. (A–F) Metaphase chromosomes from the indicated genotypes of MEFs. Chromosomal breaks (black arrowhead in B), sister chromatid exchanges (white arrowheads in C and D), and radial chromosomes (arrow in E and F) are observed in *Fancm* mutant MEFs, but not wild type MEFs (A).
(TIF)

Figure S2 Representative images for PGC quantification and proliferation in E13.5 embryonic gonads. Wild type (A, C) and *Fancm^C4/C4^* (B, D) male (A, B) and female (C, D) gonads are immunolabeled for Stella, a PGC marker, in red and BrdU in green.
(PDF)

Figure S3 TUNEL assay of PGCs in E12.5 gonads. Wild type (A, C) and *Fancm^C4/C4^* (B, D) female (A, B) and male (C, D) gonads are immunolabeled for Stella, a PGC marker, in red and TUNEL in green. (E) *Atm^−/−^* testis was used as a positive control for TUNEL signal (green).
(TIF)

Table S1 Viability of *Fancm* mutant mice.
(DOCX)

Table S2 Tumor Frequency of *Fancm* mutants.
(DOCX)

Table S3 Histopathology of *Fancm* mutant mice.
(XLS)
