# Correction: Hypersensitivity of Primordial Germ Cells to Compromised Replication-Associated DNA Repair Involves ATM-p53-p21 Signaling

**DOI:** 10.1371/journal.pgen.1004730

**Published:** 2014-09-12

**Authors:** The PLOS Genetics Staff

The following Acknowledgements are missing from the manuscript. They should read “We thank Gabriel Balmus for participating in the mapping and sequencing of Chaos4, and Robert Munroe for the ES cell microinjections.”

## References

[1] Luo Y, Hartford SA, Zeng R, Southard TL, Shima N, et al (2014) Hypersensitivity of Primordial Germ Cells to Compromised Replication-Associated DNA Repair Involves ATM-p53-p21 Signaling. PLoS Genet 10(7): e1004471 doi:10.1371/journal.pgen.1004471 25010009 10.1371/journal.pgen.1004471 PMC4091704

