# Supplementary figures and images for: Hypersensitivity of Primordial Germ Cells to Compromised Replication-Associated DNA Repair Involves ATM-p53-p21 Signaling
Source: PLoS Genet. 2014 Jul 10;10(7):e1004471. doi: 10.1371/journal.pgen.1004471 (PMC4091704; doi:10.1371/journal.pgen.1004471)

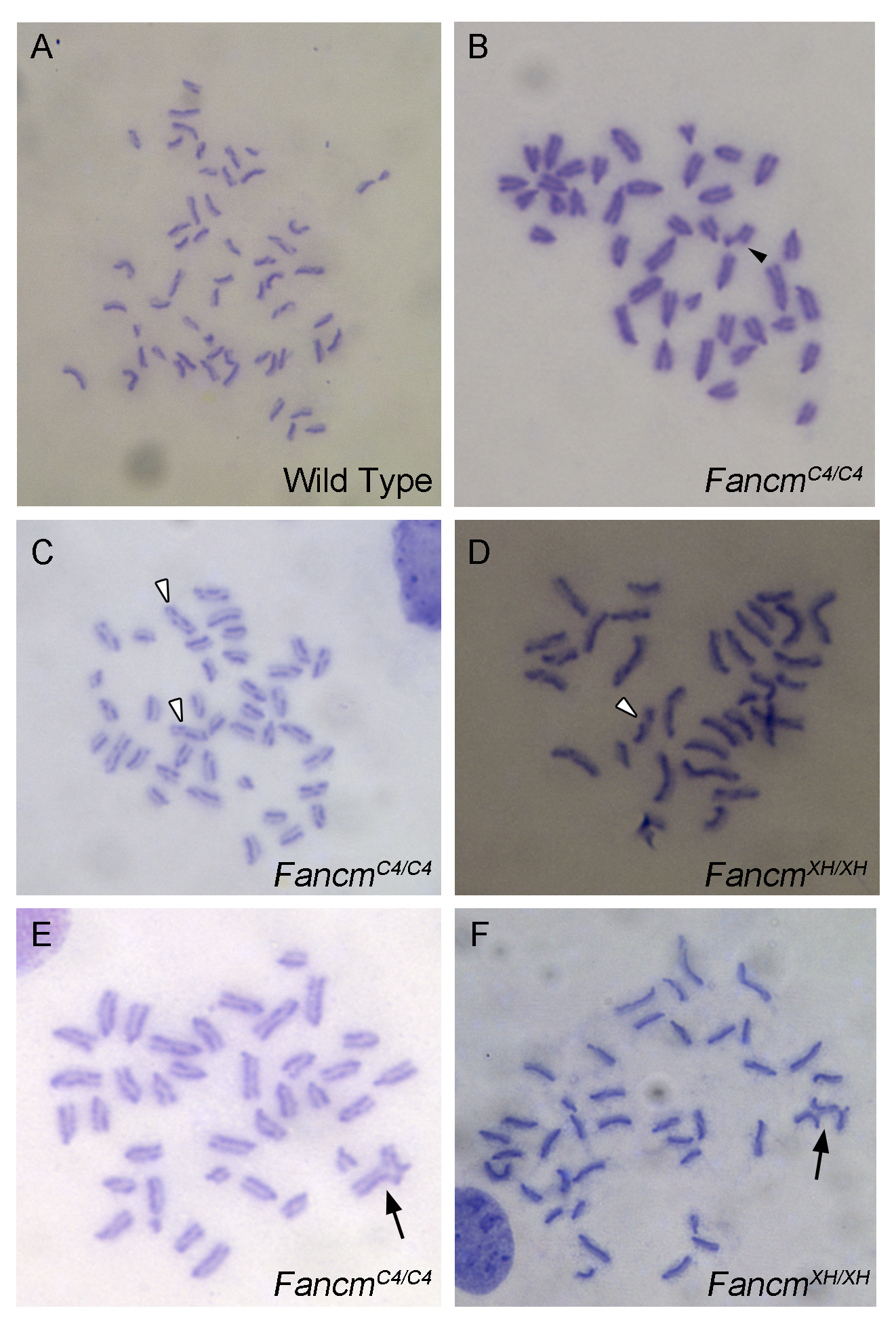

Supplement: Figure S1 — Chromosomal instability in Fancm mutant MEFs. (A–F) Metaphase chromosomes from the indicated genotypes of MEFs. Chromosomal breaks (black arrowhead in B), sister chromatid exchanges (white arrowheads in C and D), and radial chromosomes (arrow in E and F) are observed in Fancm mutant MEFs, but not wild type MEFs (A). (TIF) [file pgen.1004471.s001.tif]

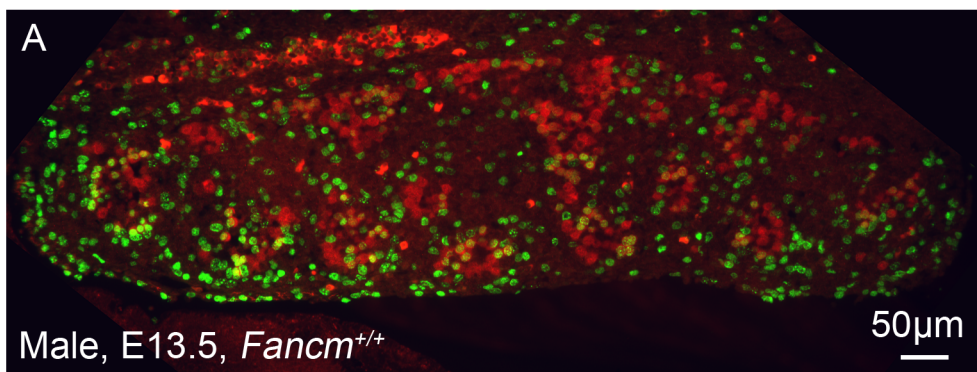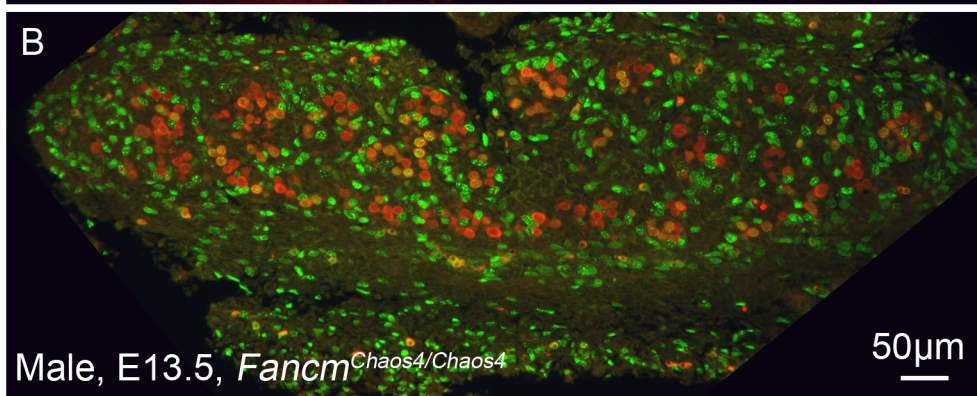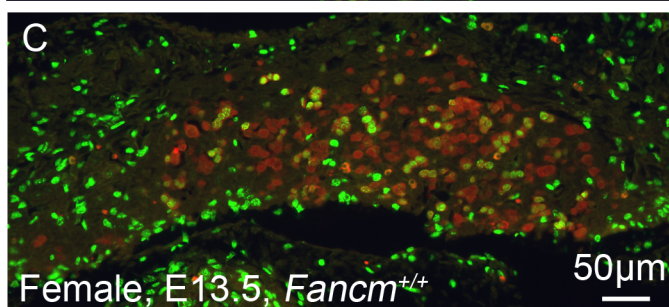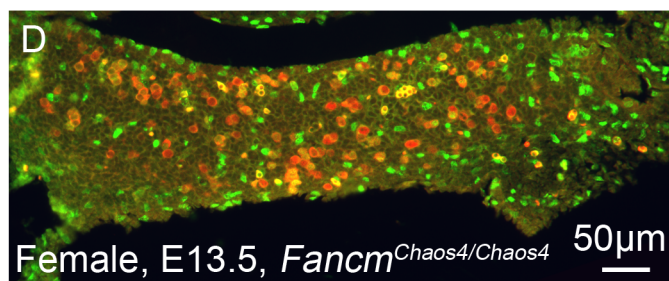

Supplement: Figure S2 — Representative images for PGC quantification and proliferation in E13.5 embryonic gonads. Wild type (A, C) and FancmC4/C4 (B, D) male (A, B) and female (C, D) gonads are immunolabeled for Stella, a PGC marker, in red and BrdU in green. (PDF) [file pgen.1004471.s002.pdf]

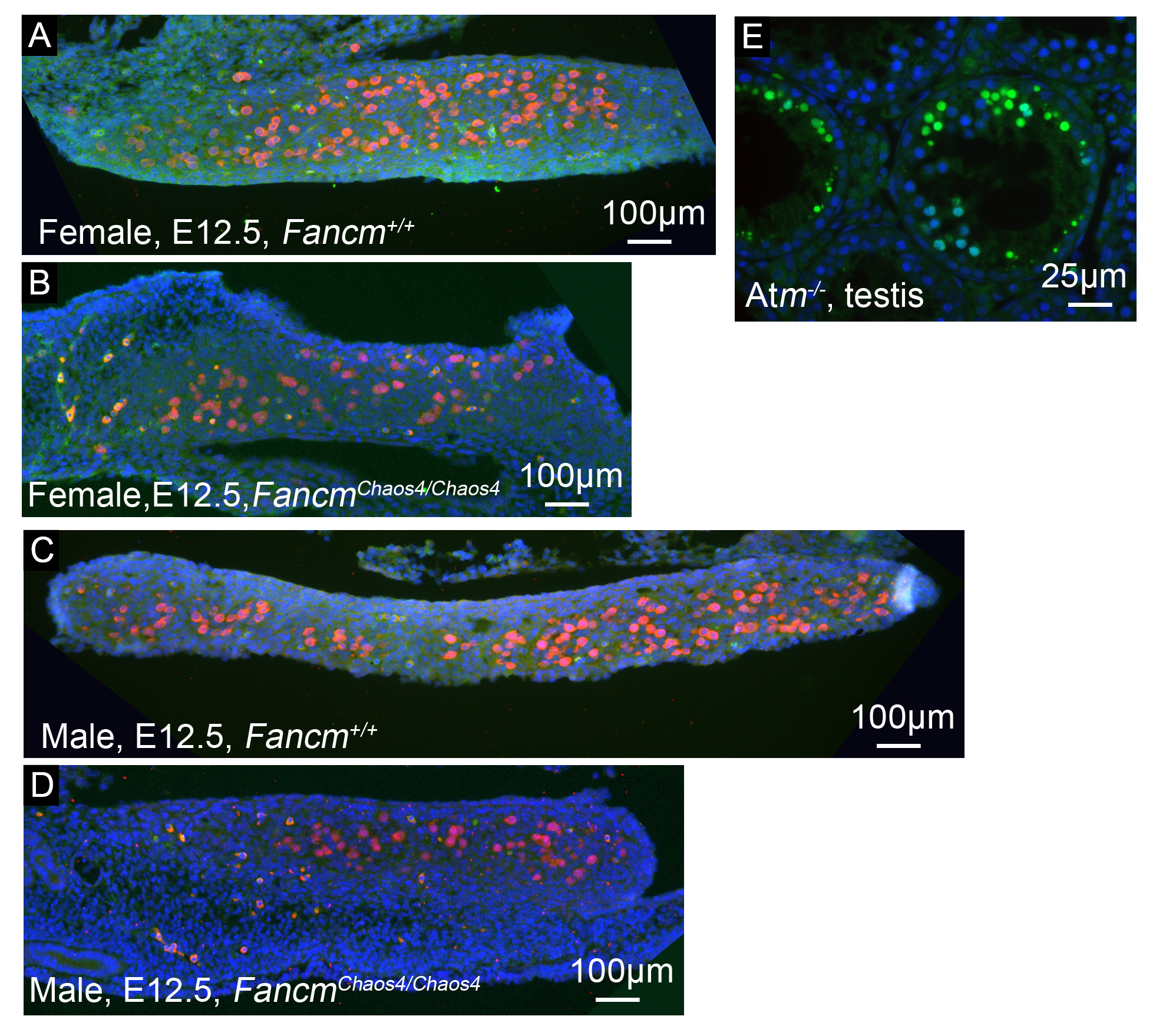

Supplement: Figure S3 — TUNEL assay of PGCs in E12.5 gonads. Wild type (A, C) and FancmC4/C4 (B, D) female (A, B) and male (C, D) gonads are immunolabeled for Stella, a PGC marker, in red and TUNEL in green. (E) Atm−/− testis was used as a positive control for TUNEL signal (green). (TIF) [file pgen.1004471.s003.tif]
