# Supplementary material for: Hypersensitivity of Primordial Germ Cells to Compromised Replication-Associated DNA Repair Involves ATM-p53-p21 Signaling
Source: PLoS Genet. 2014 Jul 10;10(7):e1004471. doi: 10.1371/journal.pgen.1004471 (PMC4091704; doi:10.1371/journal.pgen.1004471)
Supplement: Table S1 — Viability of Fancm mutant mice. (DOCX) [file pgen.1004471.s004.docx]

**Table S1. Viability of *Fancm* mutant mice**

| *Fancm^+/C4^*× *Fancm^+/C4^* | | | | | | |
| --- | --- | --- | --- | --- | --- | --- |
|  | Male | | Female | | Total | |
|  | Observed | Expected | Observed | Expected | Observed | Expected |
| *+/+* | 49 | 44 | 44 | 44 | 93 | 88 |
| *Fancm^+/C4^* | 81 | 88 | 84 | 88 | 165 | 175 |
| *Fancm^C4/C4^* | 40 | 44 | 52 | 44 | 92 | 88 |
| χ^2^ test | p=0.48 | | p=0.43 | | p=0.56 | |
|  |  |  |  |  |  |  |
|  |  |  |  |  |  |  |
| *Fancm^+/XH^*× *Fancm^+/XH^* | | | | | | |
|  | Male | | Female | | Total | |
|  | Observed | Expected | Observed | Expected | Observed | Expected |
| *+/+* | 12 | 8 | 11 | 8 | 21 | 16 |
| *Fancm^+/XH^* | 10 | 16 | 15 | 16 | 25 | 33 |
| *Fancm^XH/XH^* | 12 | 8 | 5 | 8 | 17 | 16 |
| χ^2^ test | p=0.05 | | p=0.31 | | p=0.10 | |
|  |  |  |  |  |  |  |
|  |  |  |  |  |  |  |
| *Fancm^+/C4^*× *Fancm^+/XH^* | | | | | | |
|  | Male | | Female | | Total | |
|  | Observed | Expected | Observed | Expected | Observed | Expected |
| *+/+* | 4 | 3 | 4 | 3 | 6 | 6 |
| *Fancm^+/C4^* | 5 | 3 | 2 | 3 | 3 | 6 |
| *Fancm^+/XH^* | 1 | 3 | 3 | 3 | 8 | 6 |
| *Fancm^C4/XH^* | 2 | 3 | 4 | 3 | 8 | 6 |
| χ^2^ test | p=0.36 | | p=0.83 | | p=0.44 | |
