# Supplementary material for: Hypersensitivity of Primordial Germ Cells to Compromised Replication-Associated DNA Repair Involves ATM-p53-p21 Signaling
Source: PLoS Genet. 2014 Jul 10;10(7):e1004471. doi: 10.1371/journal.pgen.1004471 (PMC4091704; doi:10.1371/journal.pgen.1004471)
Supplement: Table S2 — Tumor Frequency of Fancm mutants. (DOCX) [file pgen.1004471.s005.docx]

**Table S2**. Tumor Frequency of *Fancm* mutants.

| Tumor Frequency-Females | | |
| --- | --- | --- |
| Wild type | 0/28 (44-80wks) | 0.00% |
| *Fancm^+/C4^* | 9/27 (44-80wks) | 33.33% |
| *Fancm^C4/C4^* | 15/26 (44-80wks) | 57.69% |
| *Fancm^XH/XH^* | 0/5 (28-52wks) | 0.00% |
|  |  |  |
| Tumor Frequency-Males | | |
| Wild type | 4/45 (50-85wks) | 9.00% |
| *Fancm^+/C4^* | 15/36 (60-90wks) | 41.67% |
| *Fancm^C4/C4^* | 8/17 (70-80wks) | 47.06% |
| *Fancm^XH/XH^* | 0 out of 8 (12-78wks) | 0.00% |
